# Cerebral phaeohyphomycosis due to *Rhinocladiella mackenziei* in an immunocompetent patient: A case report and review of literature

**DOI:** 10.18502/CMM.6.3.4497

**Published:** 2020-09

**Authors:** Muhammad Zain Mushtaq, Saad Bin Zafar Mahmood, Nosheen Nasir, Malik Saad Rashid, Memoona Irshad, Kiren Habib, Iffat Khanum

**Affiliations:** 1 Department of Medicine, Aga Khan University Hospital, Stadium Road, Karachi, Pakistan

**Keywords:** Brain abscess, Cerebral phaeohyphomycosis, Fungal, Pakistan, *Rhinocladiella mackenziei*

## Abstract

**Background and Purpose:**

*Rhinocladiella mackenziei* is a neurotropic fungus, which can cause devastating intracerebral infections with up to 100% fatality rate. It is difficult to isolate this fungus in laboratory as it grows slowly and requires diagnostic skills.

**Case report:**

A 42-year-old Pakistani man presented with headache, facial numbness, progressive upper limb weakness, and dysarthria. Magnetic resonance imaging of the brain showed a space-occupying lesion in the basal ganglia region. The patient underwent supratentorial craniotomy for biopsy and excision. Histopathology of the specimen revealed granulomatous inflammation with abscess formation. Periodic acid- Schiff special stains highlighted the presence of numerous septate fungal hyphae. The results revealed the growth of dematiaceous fungi, which were morphologically classified as *R. mackenziei*. The patient is currently stable and is being on amphotericin and posaconazole, along with neurorehabilitation therapy.

**Conclusion:**

*Rhinocladiella mackenziei* brain abscess is a devastating infection with significant mortality. This condition should be suspected in patients with brain abscess from high endemic areas.

## Introduction

Derebral phaeohyphomycosis is an infection caused by dematiaceous (darkly pigmented) fungi resulting in hyphae and sometimes yeast-like cells in tissues [ 1 ]. This condition is

characterized by the development of black necrotic brain tissue, black pus, and cerebrospinal fluid [ 2 ]. The common organisms causing this disease include *Cladophialophora bantiana, Exophiala dermatitidis,* and *Rhinocladiella mackenziei* [ 1 ]. *Rhinocladiella mackenziei* is a neurotropic fungus, a member of the fungal family Herpotrichiellaceae (order Chaetothyriales). This species is unique in its ability to cause a devastating cerebral infection with almost 100% fatality rate despite aggressive treatment with surgery and intensive fungal therapy [ 3 - 5 ]. To the best of our knowledge, no environmental source has been established for this organism so far [ 1 , 2 ]. The current paper reports a life-threatening case of cerebral phaeohyphomycosis due to *R. mackenziei* in an immunocompetent male.

## Case report

A 42-year-old man, from Baluchistan, Pakistan, with no significant prior comorbidity, presented with a 5 to 7-day history of left frontal and retro-orbital headache, left facial numbness, right upper limb weakness, and speech difficulty. He had no prior history of immunosuppression or weight loss and had no recent travel. Prior to these complaints, he had a one-month history of injudicious detention in prison with reported torture. On examination, he was afebrile, alert, and oriented to time, place, and person. He had dysarthria, left-sided facial weakness, and reduced power in the right upper limb.

**Figure 1:**
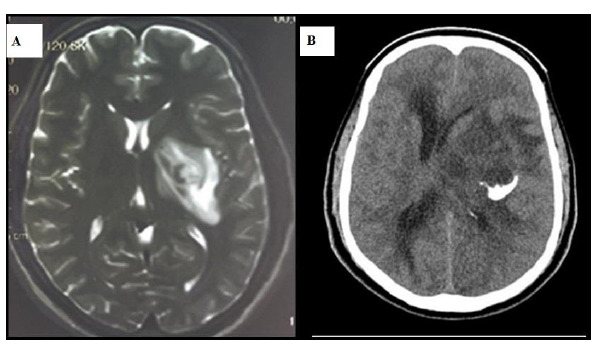
A) Magnetic resonance imaging of the brain with contrast, B) Computed tomography scan of the head without contrast

Magnetic resonance imaging (MRI) of the brain with contrast showed a space-occupying lesion in the basal ganglia region (Figure 1A). Other investigations performed at admission are summarized in Table 1. The patient underwent supratentorial craniotomy for the excision of the left thalamic space-occupying lesion, revealing thick-walled cavity with gross purulent (greenish-brown) discharge. He was subjected to maximum excision, and the tissue specimen was sent for histopathological and microbial examinations. Apart from becoming aphasic, the patient recovered rapidly in the postoperative period.

**Table 1:** Summary of the investigations performed upon patient admission

Tests	Result (mg/dL unless specified)
Hemoglobin	15.0 g/dL
White cell count	11.6 x 10E9/L
Neutrophils	82.2%
Lymphocytes	11.0%
Platelets	271 x 10E9/L
Blood urea nitrogen	18
Creatinine	0.8
Sodium	136 mmol/L
Potassium	4.1 mmol/L
Chloride	102 mmol/L
Bicarbonate	27.1 mmol/L
Calcium	9.0
Magnesium	2.0
Erythrocyte sedimentation rate	3 mm/1^st^ h
C- reactive protein	0.14
Human immunodeficiency virus assay	Negative
β-d-Glucan	<7.812 pg/ml
Galactomannan	0.115
Blood culture	Negative

**Figure 2:**
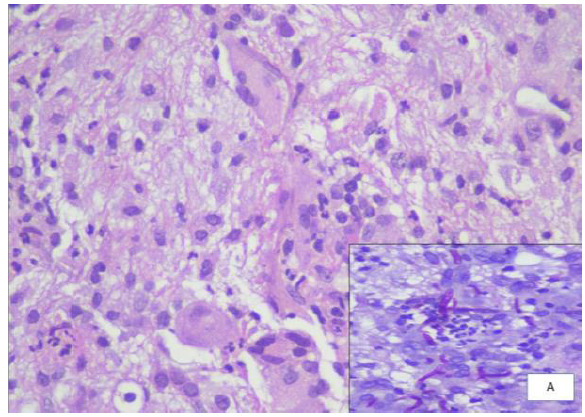
Histopathological slide stained with haematoxylin and eosin (X40) and periodic acid Schiff special stain (X40)

The tissue sample was mixed with 10% KOH and visualized at 10× and 40× magnification. It was initially examined with hematoxylin and eosin stain revealing glial parenchyma with chronic granulomatous inflammation, showing the collection of histiocytes and multinucleated giant cells (Figure 2). Further staining with periodic acid-Schiff highlighted the presence of moderate to darkly pigmented septate hyphae with no yeast formation (Figure 2A).

**Figure 3:**
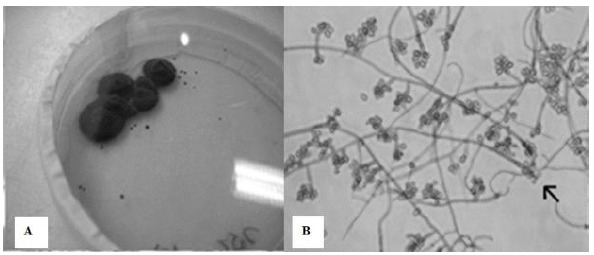
A) Mature colonies of *Rhinocladiella mackenziei* and B) photomicrograph of a slide culture harvested over 3 weeks

The patient was empirically treated with intravenous (IV) voriconazole (200 mg) 12 h after loading a dose of 400 mg every 12 h. However, he developed worsening drowsiness and complete right- sided hemiplegia. A non-contrast computed tomography (CT) scan of the head showed signs of mass effect with a midline shift of 10 mm towards the right side. In view of disease progression, voriconazole was switched to IV amphotericin B deoxycholate (1 mg/kg/day). Furthermore, 3% hypertonic saline and dexamethasone were continued to reduce intracranial pressure. In order to determine the microbiological identification of the organism, the brain tissue specimen was inoculated on sheep blood agar (Merck, Germany), Saboraud’s dextrose agar (Merck, Germany), and potato dextrose agar (Merck, Germany). The plates were incubated at 27oC and 37oC and were observed daily. After 5 weeks, the growth of black yeast-like fungi was observed (Figure 3A). Slide culture was prepared on malt extract agar (Merck, Germany), and the *Rhinocladiella*-type sporulation (“Mickey Mouse” appearance) of fungi were seen (Figure 3B), which were morphologically classified as *R. mackenziei*.

Since no facility was available at our institute for the molecular testing of rare fungi, the diagnosis of cerebral phaeohyphomycosis secondary to *R. mackenziei* was made on the basis of morphological characteristics. It was not possible to perform antifungal susceptibility testing. The patient was started on oral posaconazole (300 mg) once a day, in addition to IV amphotericin B deoxycholate. Despite being kept on hyperosmolar therapy and antifungals, the patient condition deteriorated after 18 days and the Glasgow coma scale (GCS) dropped from 11 to 4. Urgent non-contrast CT scan of the head showed an increased midline shift of up to 11.2 mm towards the right side resulting in diffuse cerebral edema and left uncal herniation (Figure 1B).

The patient underwent emergent decompressive craniectomy. Postoperatively, the GCS improved to 11/15, and he was able to localize from the left side. Along with amphotericin B and posaconazole, a therapeutic trial of flucytosine (3 gm) was also initiated every 6 h but later discontinued as there was no meaningful improvement, and financial constraints led to difficulty in procurement. Currently, the patient has recovered with neurologic sequelae and is still undergoing treatment with IV amphotericin B and oral posaconazole, along with neurorehabilitation therapy.

**Ethical considerations**

Informed consent was obtained from the next of kin, and the study was approved as an exemption by the Ethical Review Committee of Aga Khan University Hospital, Karachi, Pakistan (Reference # 2019-1792- 4664).

## Discussion

Herein, we presented a case of cerebral phaeohyphomycosis caused by *R. mackenziei* in an immunocompetent male who recovered with neurological sequelae after extensive surgical and antifungal treatment, along with neurorehabilitation. *Rhinocladiella mackenziei* has been a recognized cause of cerebral phaeohyphomycosis [ 3 ]. Literature review shows that between 1983 and 2017, approximately 31 cases of brain abscess were reported to be secondary to *R. mackenziei* [ 6 ]. Infections have been predominantly reported from the Middle East and Southeast Asian regions with tropical and dry climate [ 3 ]. Sporadic cases in Europe or North America have also involved immigrants from the Middle East suggesting that race is also an important factor [ 4 , 7 ]. The results of our case, who was from Baluchistan, were similar to the findings reported in the literature from Pakistan [ 8 ].

Our patient had no known immunodeficient state. Based on the literature, the incidence of this disease is now on a growing trend in immunocompetent individuals [ 6 , 9 ]. Nonetheless, immunodeficient states, like solid organ transplant recipients, connective tissue disorders, chronic liver disease, or prolonged steroid use, have long been implicated in individuals predisposing to this infection [ 2 ]. The mode of transmission is mostly through bloodstream or lymphatics. However, the direct inoculation of the pathogen to the contiguous site (due to trauma) is also one of the modes of transmission and may have been the mode of transmission in our patient as he had injuries from trauma afflicted during his imprisonment [ 10 ].

Clinical presentation of *R. mackenziei* infections has a vast spectrum, ranging from pneumonia to fatal brain abscess and disseminated infections [ 1 , 2 ]. It is impossible to establish a diagnosis solely based on clinical presentations as symptoms are typical of any lesion in the brain, such as fevers, seizures, behavioral changes, headaches, and focal and hemiparesis or hemisensory loss [ 1 , 11 ]. Similar findings were observed in our case as well. Discoloration of cerebrospinal fluid from green to brown is a distinctive feature of *R. mackenziei* if meninges are involved [ 2 ].

Brain abscess is a pathognomonic feature of phaeohyphomycosis. In a study, 51.6% of patients had a single lesion, while 48.4% of them had multiple brain lesions [ 6 ]. Immunocompetent individuals mostly present with a single lesion, while multiple lesions can occur in immunocompromised hosts. Typical radiological findings of MRI were suggestive of a ring-enhancing lesion on T1-weighted images, hyperintensity on diffusion-weighted images, and low- to high-signal intensity on apparent diffusion coefficient sequence [ 1 , 12 ]. Our patient had a single ring-enhancing lesion in the basal ganglia region on T1-weighted images, which also showed substantial hyperintensity on T2-weighted images, suggesting vasogenic edema. The differential diagnoses for a cerebral lesion in our case based on history and examination included neoplasm, multiple sclerosis, sarcoidosis, and infectious diseases, like tuberculosis, bacterial abscess, and other dematiaceous filamentous fungal infections.

The classical histopathological features of cerebral phaeohyphomycosis are the presence of dematiaceous fungal material, granulomatous inflammation, and vasculitis with giant cell infiltration [ 2 ]. Histopatho- logical examination in our case also depicted granuloma; however, there was no evidence of vasculitis with giant cell infiltration in our case as our patient had no history of mycotic thrombosis and subsequent artery infarction.

The latest joint clinical guidelines from ESCMID/ ECMM for cerebral phaeohyphomycosis recommended a combination of antifungal therapy with the complete excision of brain abscess [ 6 , 9 ]. However, overall mortality remains near 100%, even with both surgical debridement and antifungal therapy, while the mean survival from the onset of treatment is reported to be 4.7 months [ 6 ]. Our patient underwent neuronavigation-guided supratentorial craniotomy and maximal excision. Later on, when the health condition of the patient deteriorated, he was also subjected to decompressive craniectomy. Authors agree that whenever excision is possible, it should be preferred over surgical procedure [ 7 , 10 , 11 ]. The recovery of our patient also favors this argument.

The most commonly used antifungal agents are amphotericin B, flucytosine, posaconazole, voriconazole, and itraconazole. Both single and combination therapies have been suggested [ 1 ]. Antifungal susceptibility should be ascertained at the onset and during the course of therapy to document resistance. Our patient initially received voriconazole but was shifted to amphotericin once the clinical condition deteriorated. Once the identification of *R. mackenziei* was made, the patient was given the trials of flucytosine (no benefit) and posaconazole.

The two latest review article on R. mackenziei have conflicting results regarding which azoles to use [ 1 , 6 ]. Yusupov et al. suggested that posaconazole is equal if not better than voriconazole for the treatment of cerebral phaeohyphomycosis due to *R. mackenziei* [ 1 ]. Posaconazole has also been favored by Al-Abdely et al. [ 13 ], Hardman et al. [ 11 ], and Pitisuttihum et al. [ 14 ]. However, Mohammadi et al. introduced voriconazole to be more effective than Posaconazole [ 6 ]. This argument is strengthened by the latest joint clinical guidelines from ESCMID/ECMM for cerebral phaeohyphomycosis, indicating the higher efficiency of voriconazole in penetrating the brain tissue. There are also recommendations regarding the use of flucytosine, both as single therapy [ 9 ] and in combination with amphotericin [ 15 ]. However, flucytosine and fluconazole have high minimum inhibitory concentrations (MICs); therefore, they were concluded to be ineffective in a study [ 16 ]. This clearly shows that there is yet to be a consensus about optimal antifungal medications.

Our diagnostic workup was limited due to the lack of resources available for molecular and susceptibility testing of rare fungi. In a previous, funded study from our institute, the molecular identification of *R. mackenziei* was performed at the Centers for Disease Control and Prevention [ 8 ]. In another recent case report, the diagnosis of cerebral phaeohyphomycosis was made on the basis of only morphological characteristics due to the non-availability of molecular testing [ 17 ]. Our patient was treated empirically and responded well to a combination of amphotericin and posaconazole. The patient was also discharged on posaconazole with considerable improvement in GCS; however, he still had right-sided weakness and aphasia. Significant neurological sequelae have been observed in almost all of the cases who have recovered [ 11 , 13 ].

## Conclusion

*Rhinocladiella mackenziei* brain abscess is a devastating infection with significant morbidity and mortality. This condition should be suspected in patients presenting with brain abscess from high endemic areas.
